# Semi-Interpenetrating Polymer Networks Based on Cyanate Ester and Highly Soluble Thermoplastic Polyimide

**DOI:** 10.3390/polym11050862

**Published:** 2019-05-13

**Authors:** Jingfeng Liu, Weifeng Fan, Gewu Lu, Defeng Zhou, Zhen Wang, Jingling Yan

**Affiliations:** 1 College of Chemical Engineering, Changchun University of Technology, Changchun 130012, China; austin@ciac.ac.cn; 2 Laboratory of Polymer Composites Engineering, Changchun Institute of Applied Chemistry, Chinese Academy of Science, Changchun 130022, China; fwf@ciac.ac.cn (W.F.); wz@nimte.ac.cn (Z.W.); 3 Aerospace Research Institute of Special Materials & Technology, Beijing 100074, China; lugw03@126.com; 4 Ningbo Institute of Material Technology & Engineering, Chinese Academy of Science, Ningbo 315201, China

**Keywords:** thermoplastic polyimide, cyanate ester, semi-interpenetrating polymer network, toughness, dielectric properties

## Abstract

Thermoplastic polyimide (TPI) was synthesized via a traditional one-step method using 2,3,3′,4′-biphenyltetracarboxylic dianhydride (3,4′-BPDA), 4,4′-oxydianiline (4,4′-ODA), and 2,2′-bis(trifluoromethyl)benzidine (TFMB) as the monomers. A series of semi-interpenetrating polymer networks (semi-IPNs) were produced by dissolving TPI in bisphenol A dicyanate (BADCy), followed by curing at elevated temperatures. The curing reactions of BADCy were accelerated by TPI in the blends, reflected by lower curing temperatures and shorter gelation time determined by differential scanning calorimetry (DSC) and rheological measurements. As evidenced by scanning electron microscopy (SEM) images, phase separation occurred and continuous TPI phases were formed in semi-IPNs with a TPI content of 15% and 20%. The properties of semi-IPNs were systematically investigated according to their glass transition temperatures (*T_g_*), thermo-oxidative stability, and dielectric and mechanical properties. The results revealed that these semi-IPNs possessed improved mechanical and dielectric properties compared with pure polycyanurate. Notably, the impact strength of semi-IPNs was 47%–320% greater than that of polycyanurate. Meanwhile, semi-IPNs maintained comparable or even slightly higher thermal resistance in comparison with polycyanurate. The favorable processability and material properties make TPI/BADCy blends promising matrix resins for high-performance composites and adhesives.

## 1. Introduction

Cyanate ester (CE) resins have been widely utilized as matrix resins in electronic and aerospace industries thanks to several distinct advantages over conventional epoxy resins, including low dielectric constant and loss factor, high glass transition temperature (*T_g_*), reduced moisture absorption and outgassing, good processability, compatibility with various substrates and reinforcements, and favorable mechanical properties [1,2,3,4]. Polycyanurates, the cured products of CE resins, possess higher fracture toughness in comparison with other traditional thermosets (epoxy, bismaleimide, etc.). Nevertheless, toughening of CE resins is still indispensable for some highly demanding applications, especially fiber-reinforced composites. In addition, catalysts, typically comprising metal salts and phenols, are usually exploited to lower the curing temperatures of CE resins [5,6].

Blending with other reactive or nonreactive resins is an effective approach to toughening CE resins. A diversity of thermosetting resins (such as epoxides, bismaleimides (BMI), and imide oligomers) has been investigated for the modification of CE resins [7,8,9,10,11]. CE resins can copolymerize with epoxy resins, affording thermosets with enhanced fracture toughness [12,13]. However, the thermal, mechanical, and dielectric properties of the resulting polymers are significantly compromised due to the formation of oxazolidinone moieties. On the contrary, CE cannot directly react with BMI or (phenyl)ethynyl-end-capped imide oligomers. This issue could be addressed by introducing allyl-containing chain extenders, such as 2,2′-diallylbisphenol A (DABPA) [14,15]. Meier et al. prepared sequential interpenetrating polymer networks (IPNs) from novolac-type CE and phenylethynyl-terminated imide oligomer (PETI) with the aid of DABPA. The *T_g_* and degradation temperatures were increased with the inclusion of PETI [16]. Our group demonstrated that IPNs based on bisphenol A dicyanate (BADCy) and ethynyl-terminated imides displayed much higher mechanical properties but slightly higher thermal properties compared with pure polycyanurates [17]. In addition, BMI/CE blends possessed the merits of the two individual resins and were commercialized by Mitsubishi Gas Chemical Company, Inc.

Blending with high-performance engineering plastics is an alternative methodology for toughening CE resins. Woo et al. investigated the influence of thermoplastic contents on the phase behavior and, consequently, the fracture toughness of modified thermosets derived from bisphenol E dicyanate and engineering plastics (polyethersulfone (PES) and polyetherimide (PEI)). Continuous thermoplastic phases were formed at high PES loading (>10%), and thus, the fracture toughness of the semi-IPNs were remarkably increased [18]. Iijima et al. modified BADCy with copolyimides derived from 3,3,4′,4′-biphenyltetracarboxylic dianhydride (4,4′-BPDA), 4,4′-(hexafluoroisopropylidene)diphthalic anhydride (6FDA), and 4,4′-bis(*m*-aminophenoxy) diphenylsulfone. It was observed that polyimides with low compatibility with CE resins were more efficient for improving the mechanical properties of the resultant thermosets. With the addition of 15% copolyimides, the fracture toughness was increased by 65% without significantly diminishing other thermal and mechanical properties [19]. Polyimide/polysiloxane block copolymers were explored as CE modifiers by Liang and coworkers. They demonstrated that the tensile strength, modulus, and fracture toughness could be markedly enhanced with 5% inclusion of the abovementioned block copolymers [20]. Liang et al. also modified CE resins with highly soluble phenolphthalein-based PES (PESC), which facilitated the solventless formulation of the blends. Considerable improvements were achieved in terms of impact strength, flame retardancy, and dielectric constant and loss factor when compared with pure polycyanurates. However, the majority of thermoplastics for CE modifications possessed a *T_g_* of less than 250 °C because of the presence of a flexible ether linkage, which could downgrade the *T_g_* and high-temperature modulus of the resultant thermosets [21].

**Scheme 1:**
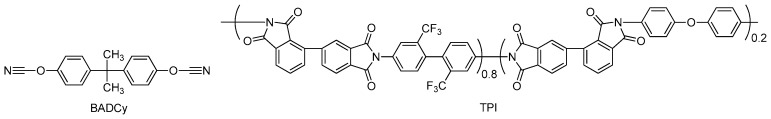
Structures of bisphenol A dicyanate (BADCy) and thermoplastic polyimide (TPI).

In this work, thermoplastic polyimide (TPI) simultaneously possessing a high *T_g_* (360 °C) and excellent solubility (>20% in BADCy) was prepared using 2,3,3′,4′-biphenyltetracarboxylic dianhydride (3,4′-BPDA), 4,4′-oxydianiline (4,4′-ODA), and 2,2′-bis(trifluoromethyl)benzidine (TFMB) as the monomers (Scheme 1). The good solubility of TPI in BADCy allowed the formulation of TPI/BADCy blends without the involvement of any organic solvents. The influence of blend compositions on the processability of the resins and the material properties of the semi-IPNs were also systematically investigated.

## 2. Experimental

### 2.1. Materials 

BADCy was provided by Yangzhou Techia Material Co., Ltd. (Yangzhou, China). 3,4′-BPDA, 4,4′-ODA, and TFMB were purchased from Changzhou Sunlight Pharmaceutical Co., Ltd. (Changzhou, China). All other reagents were obtained from Tianjin Tiantai Fine Chemical Industry Co., Ltd. (Tianjin, China). All chemicals were used as received unless specified. 

### 2.2. Synthesis of TPI

3,4′-BPDA (29.422 g, 0.100 mol), 4,4′-ODA (4.005 g, 0.020 mol), TFMB (25.618 g, 0.080 mol), and *m*-cresol (250 mL) were placed into a 500-mL, three-necked flask equipped with an overhead stirrer, a condenser, and an argon inlet and outlet. The mixture was heated at 80 °C for 10 h and then at 180 °C for 12 h in argon atmosphere. Water, generated as the byproduct of imidization, was depleted from the flask by passing an argon flow. After cooling to room temperature, the polyimide solution was poured into methanol (2 L), collected by filtration, Soxhlet extracted with methanol for 24 h, and dried in vacuum at 150 °C for 48 h to afford an off-white fibrous solid (53.4 g, yield: 96.1%). The number average molecular weight and polydispersity for this polyimide, determined by using gel permeation chromatography (GPC), were 22 kg mol^−1^ and 2.12, respectively.

### 2.3. Formulation of TPI/BADCy Semi-IPNs

TPI/BADCy blends were formulated by dissolving appropriate amounts of TPI in melting BADCy at 100 °C. The weight percentages of TPI were 3%, 5%, 10%, 15%, and 20%. The blends were accordingly designated as Blend−3, Blend−5, Blend−10, Blend−15, and Blend−20. The homogenous solution was then transferred into a preheated stainless-steel mold and degassed at 100 °C in vacuum for 3 h. The blend was then cured at 140 °C for 1 h, 180 °C for 1 h, 200 °C for 1 h, and 220 °C for 2 h to afford the corresponding semi-IPN. The polymer was then removed from the mold at 150 °C. Semi-IPNs were named by the blend compositions (e.g., Semi-IPN−10 was produced from Blend−10). 

The specimens of pure TPI were prepared by compression-molding in vacuum. TPI powder was placed into a stainless mold and heated at 300 °C for 20 min on a hot press. Then, pressure (50 MPa) was applied, and the temperature was ramped up to 420 °C in 30 min and held at 420 °C for 40 min. The shapes were cooled to 250 °C under pressure, and the pressure was subsequently released. The samples were released from the mold and stored under ambient conditions prior to characterization. 

### 2.4. Characterization

Fourier transform infrared spectroscopy (FT-IR) was carried out on a Bruker VERTEX 70 spectrometer (Bruker Co., Billerica, MA, USA). GPC was performed on a Waters 1500 GPC apparatus (Waters Co., Milford, MA) with a refractive index detector, using *N*,*N*-dimethylformamide (DMF) as the eluent and polystyrene as the standard. Differential scanning calorimetry (DSC) was recorded on a DSC Q2000 (TA Instruments, New Castle, DE, USA) in nitrogen. Thermogravimetric analysis (TGA) was carried out on a TGA Q50 (TA Instruments, New Castle, DE, USA) from 100 to 800 °C in air, with a heating rate of 10 °C min^−1^. Dynamic mechanical analysis (DMA) was performed on a Q800 DMA (TA Instruments, New Castle, DE, USA) from 50 to 400 °C with a heating rate of 3 °C min^−1^ and a frequency of 1.0 Hz. Rheological measurements were conducted on an AR2000ex rheometer (TA Instruments, New Castle, DE, USA). Tensile and flexural properties were determined on an Instron material testing system (Model 5982, Instron, Norwood, MA, USA). Unnotched impact strength was measured by using a cantilever beam impact testing machine (JJ−20, Changchun, China). Dielectric constant and loss factor were measured by using an Agilent impedance analyzer (Agilent Technologies, Santa Clara, CA, USA). SEM was conducted on a XL−30ESEM FEG microscope (Mico FEI Philips, Eindhoven, the Netherlands)) with an operating voltage of 25 kV. Semi-IPN specimens were fractured at ambient temperature, etched with dichloromethane for 48 h, and coated with a fine gold layer prior to SEM measurements.

## 3. Results and Discussion 

### 3.1. Properties of TPI/BADCy Blends

#### 3.1.1. Rheological Properties

Engineering plastics, such as PES, PEI, and PESC, are well-known toughening agents for thermosetting resins thanks to their relatively high *T_g_* and favorable mechanical properties. Nonetheless, the *T_g_* values of these polymers (200–250 °C) are significantly lower than that of BADCy-based polycyanurate (310 °C), inevitably downgrading the *T_g_* and high-temperature mechanical properties of the resultant semi-IPNs. In contrast, the majority of aromatic polyimides possess an extremely high *T_g_* (>300 °C); however, they are immiscible with CE monomers and oligomers, which imposes great difficulties in resin formulations and composite fabrications. 

**Figure 1:**
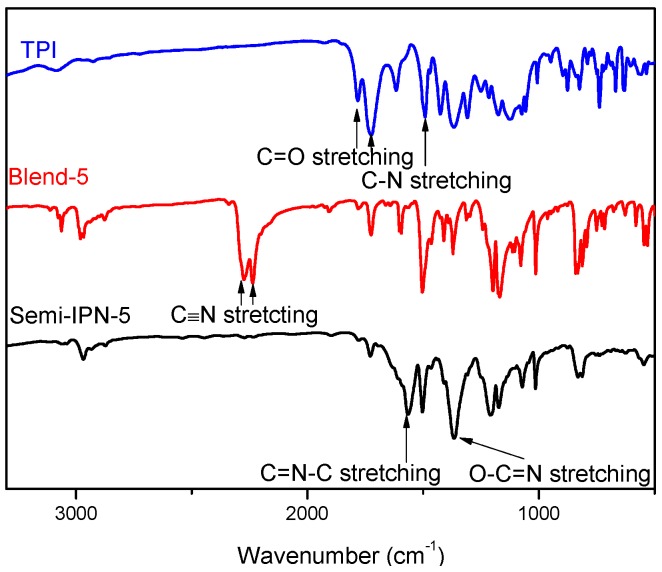
Representative FT-IR spectra of TPI, TPI/BADCy blend, and semi-interpenetrating polymer network (semi-IPN).

In this work, a TPI modifier was prepared using 3,4′-BPDA, 4,4′-ODA, and TFMB as the monomers through a conventional one-step method in *m*-cresol. Successful synthesis of the abovementioned TPI was evidenced by the characteristic absorption bands for asymmetric C=O stretching (1780 cm^−1^), symmetric C=O stretching (1720 cm^−1^), and C–N stretching (1370 cm^−1^) in FT-IR spectra (Figure 1). Due to the asymmetric architecture of 3,4′-BPDA and the fluorinated, rigid, but non-coplanar structure of TFMB, this TPI simultaneously possessed high *T_g_* and high solubility in BADCy [22,23,24,25]. Thus, TPI/BADCy blends with a TPI loading of up to 20 wt % could be easily formulated via a solvent-free procedure.

**Figure 2:**
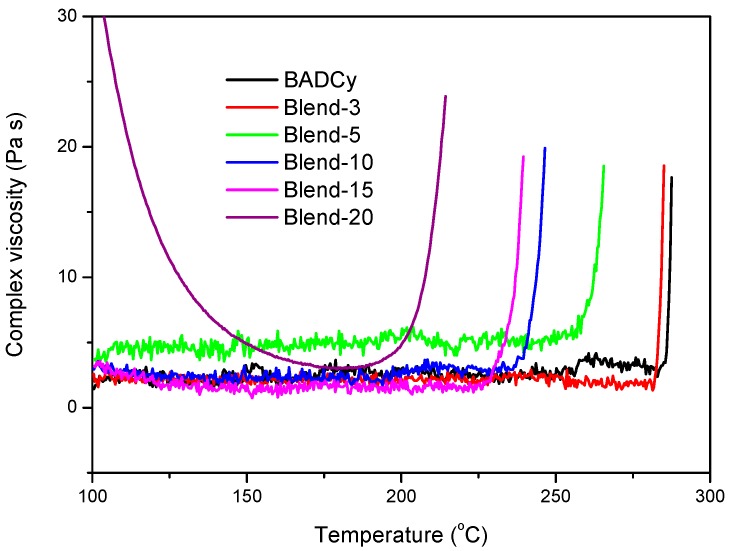
The viscosity–temperature profiles of BADCy and TPI/BADCy blends.

The curing behavior of BADCy and TPI/BADCy blends was characterized by rheological measurements. All the blends exhibited sufficiently low viscosity below 200 °C, indicating their wide processing windows (Figure 2). The viscosities then increased abruptly owing to the trimerization of -OCN functionality. The viscosity onset temperatures spanned a range of 218–286 °C, decreasing with the TPI contents. These results suggested that the cure of BADCy was catalyzed by the trace amount of amino and acid groups in TPI, which was also observed by Liang et al. [20]. To assess their suitability for prepreg manufacturing, the isothermal viscosities were measured at 100 °C. The isothermal viscosity increased with the TPI contents as expected, ranging from 0.030 to 6.366 Pa s (Table 1). Blend−20 showed the highest viscosity and the lowest viscosity onset temperature because of its highest TPI loading. All the blends possessed adequately low viscosity for prepreg preparation.

**Figure 3:**
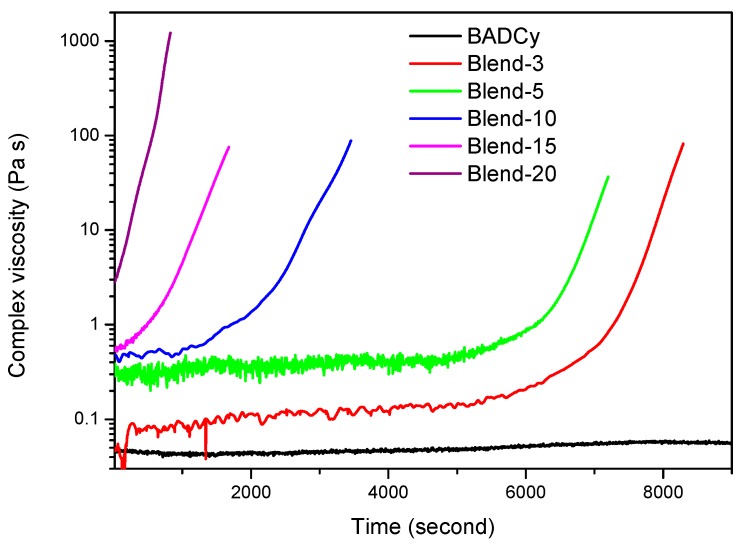
Curing behavior of BADCy and TPI/BADCy blends at 200 °C.

The gelation time of the blends was determined through isothermal viscosity measurements at 200 °C. BADCy showed stable viscosity at 200 °C, and no gelation was observed within 3 h. The gelation time of the blends was 11–131 min, decreasing with the TPI contents (Table 1 and Figure 3). These results further confirmed that TPI accelerated the polymerization of BADCy. The shorter gelation time and lower curing temperatures of the blends were conducive to the preparation of high-quality composites.

#### 3.1.2. DSC Analysis

**Figure 4:**
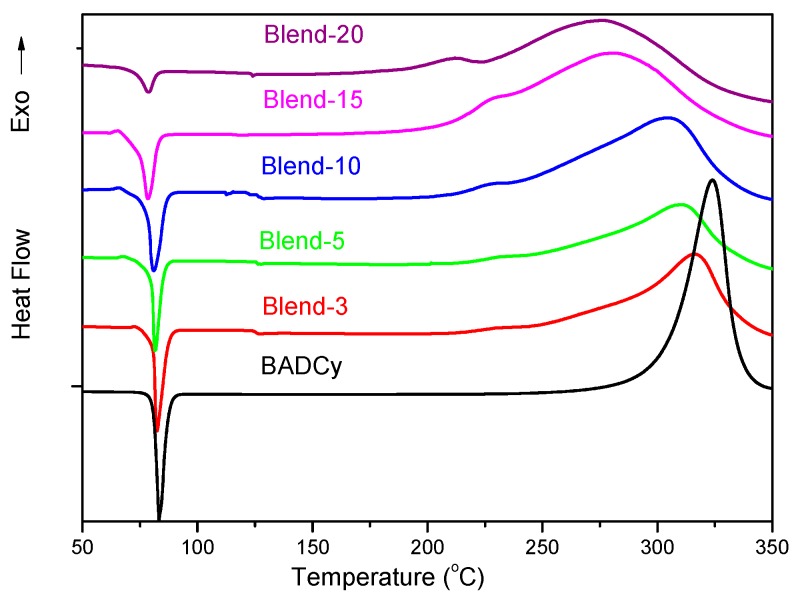
DSC traces of BADCy and TPI/BADCy blends.

The DSC traces of BADCy and TPI/BADCy blends are illustrated in Figure 4. The endothermic peaks at around 80 °C corresponded to the melting of BADCy, while the exothermic peaks located at 250–330 °C were attributed to the crosslinking of -OCN functionalities. Compared with pure BADCy, the melting peaks for all the blends tended to broaden and shift towards low temperature ranges, and the peak intensities deteriorated with the TPI contents. Moreover, similar trends were observed for the exothermic peaks. The exothermic peak temperatures (*T_p_*), corresponding to the temperature of maximum cure rate, decreased from 324 to 273 °C with an inclusion of 20% TPI (Table 1). The lower *T_p_* values and wider exothermic peaks implied that the curing conditions for all the blends were milder than that of pure BADCy, which is highly desired for the fabrication of high-quality composites. The heat of cure (Δ*H*_0_) for BADCy and TPI/BADCy blends spanned a range of 469–704 J·g^−1^, decreasing with the increase of TPI loading (Table 1). The Δ*H*_0_ values for the blends were lower than the theoretical values calculated according to the weight percentages of BADCy, suggesting that the polymerization of –OCN groups could happen to some extent during the formulation of blends.

### 3.2. Properties of Thermosets

#### 3.2.1. FT-IR Spectra

Representative spectra of TPI/BADCy blends and semi-IPNs are compared in Figure 1. The characteristic peaks at around 2230 and 2270 cm^−1^ were indicative of the existence of –OCN groups in the blends, while those at about 1720 and 1780 cm^−1^ represented the imide groups in TPI. The peaks representing –OCN functionality completely disappeared in the FT-IR spectra of semi-IPNs, confirming the completion of trimerization reactions. Furthermore, new peaks were detected at around 1565 and 1365 cm^−1^, which were relevant to the –C=N–C– and –O–C=N– stretching in triazine moieties, respectively. Meanwhile, the characteristic peaks for imide groups remained unchanged before and after cure.

#### 3.2.2. Morphology

**Figure 5:**
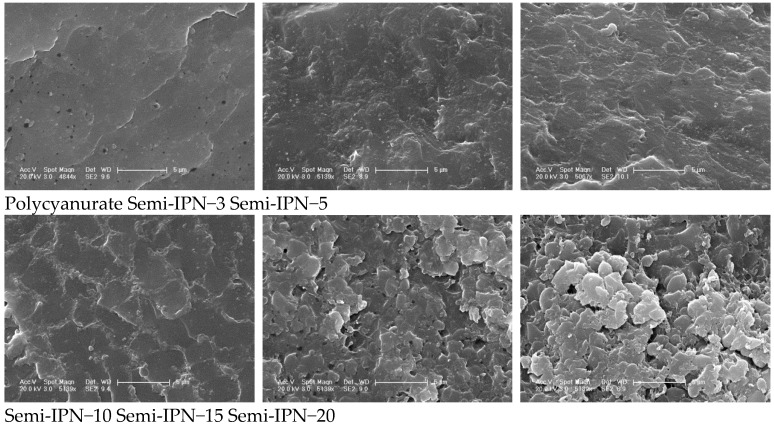
SEM images of polycyanurate and semi-IPNs.

The fractured surfaces of polycyanurate and semi-IPNs were etched by dichloromethane to remove soluble TPI and characterized through SEM. As shown in Figure 5, the solid areas corresponded to the polycyanurate phase, while the void areas represented the TPI phase removed by dichloromethane. The fracture surface of polycyanurate was smooth, exhibiting a typical brittle feature. In contrast, the fracture surfaces for the semi-IPNs were much rougher, implying improved toughness. There was no obvious phase separation observed for semi-IPNs with low TPI contents (≤10%). In contrast, Semi-IPN−15 and Semi-IPN−20 displayed apparent phase-separated morphologies, and the continuity of void areas increased with TPI contents. The co-continuous phase-separated morphological features of Semi-IPN−15 and Semi-IPN−20 could impart enhanced impact strength to the polymers.

#### 3.2.3. Dynamic Mechanical Analysis

In semi-IPN systems, thermoplastics and thermosetting components are completely miscible before cure. Thermosetting resins are converted into crosslinked networks during cure, resulting in an increase in entropy of mixing and a decrease of enthalpy of mixing. Cure-induced phase segregation tends to occur as the Gibbs free energy becomes positive. The glass transition behavior of semi-IPNs is predominated by several factors including crosslinking density, morphology, and so on. Semi-IPNs with completely phase-separated morphologies showed multiple distinct glass transitions, and the corresponding *T_g_* values were independent of blend compositions. In contrast, semi-IPNs with fully compatible compositions exhibited a single glass transition lying between those of individual polymers. For partly miscible semi-IPNs, the *T_g_* peaks tended to broaden and shift towards higher or lower temperature ranges.

**Figure 6:**
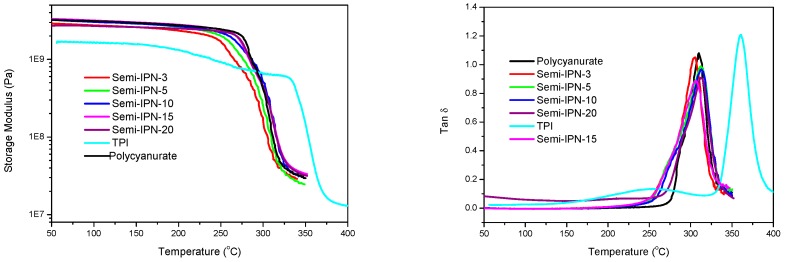
Dynamic mechanical analysis (DMA) results for polycyanurate, TPI, and semi-IPNs (Left: storage modulus versus temperature; Right: tan δ versus temperature).

The temperature dependence of storage modulus and tan δ for TPI, polycyanurate, and semi-IPNs was determined through DMA, and the results are illustrated in Figure 6. TPI exhibited two transitions at 255 and 360 °C in the tan δ curve, respectively. The major peak at 360 °C was attributed to the glass transition, while the minor peak at 255 °C was assigned to the sub-*T_g_* β-relaxation originating from the 2,2′-substituted biphenyl moiety in TMFB residue [22]. Pure polycyanurate displayed a *T_g_* of 310 °C, and the initial storage modulus was obviously higher than that of TPI. Single transitions were detected for all semi-IPNs, and the peak values were almost independent of TPI contents. Moreover, the tan δ curves became broader, with visible shoulders at a relatively low temperature end, and the peak heights were approximately proportional to the compositions of semi-IPNs. On the basis of the abovementioned results, it could be concluded that semi-IPNs in this work possessed partially (low TPI contents) or completely (high TPI contents) phase-separated morphologies. Another *T_g_*, corresponding to the primary segmental relaxation of TPI, should exist at around 360 °C. However, as the temperature exceeded the *T_g_* of polycyanurate, the storage modulus of semi-IPNs deteriorated significantly because of segmental relaxation. The dispersed TPI phase could not support itself, accounting for the absence of glass transitions in DMA curves. Generally, the incorporation of TPI did not decrease the *T_g_* and, consequently, service temperatures of the resultant thermosets and composites. The DMA results regarding semi-IPNs with low TPI contents (≤10%) seemed inconsistent with the fact that no obvious phase separation was detected in SEM measurements. This could be rationalized by the fact that the TPI domains in Semi-IPN−10 were too tiny to be observed by SEM.

#### 3.2.4. Thermo-Oxidative Stability

**Figure 7:**
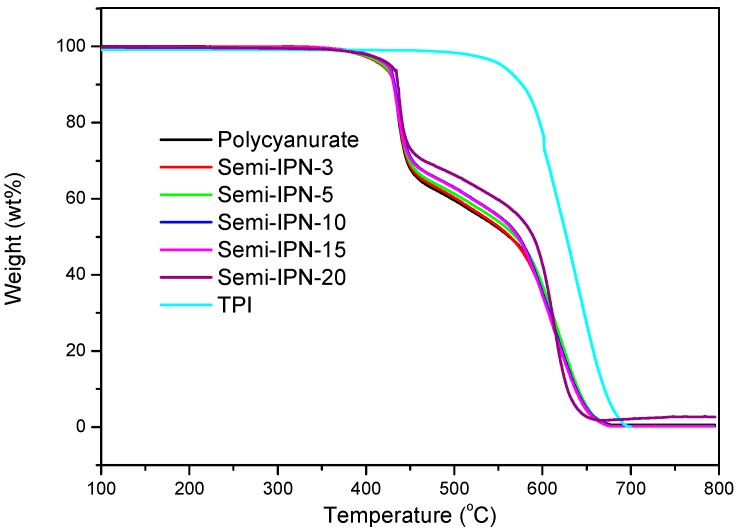
TGA curves of polycyanurate, TPI, and semi-IPNs.

The thermo-oxidative stability of all the polymers was evaluated through TGA in air. Polycyanurates and semi-IPNs showed a typical two-step decomposition pattern with a 5% weight loss temperature (*T*_5%_) of 422–429 °C (Figure 7 and Table 2). The rapid weight loss at 400–450 °C was due to the degradation of the isopropylidene and triazine segments, and then the decomposition of aromatic backbones started as the temperature exceeded 500 °C [26]. Though TPI possessed a much higher *T*_5%_ value, the incorporation of TPI did not obviously improve the thermo-oxidative stability of the semi-IPNs. The crosslinking density is a crucial factor regulating the thermal stability of thermosets, and the effect of the inclusion of TPI was cancelled by the lower crosslinking density of the semi-IPNs.

#### 3.2.5. Mechanical Properties

The mechanical properties for polycyanurate, TPI, and semi-IPNs are summarized in Table 3. Due to its thermoplastic nature and relatively high molecular weight (determined by GPC), TPI exhibited lower tensile modulus but much higher elongation at break and tensile, compression, and impact strength compared with pure polycyanurate. The impact strength of semi-IPNs was 47%–320% greater than that of pure polycyanurate, indicative of significant improvement on fracture toughness. The impact strength firstly increased and then decreased with TPI contents, with a maximum being observed at a TPI loading of 15%. The toughening effects of TPI in semi-IPNs were also reflected by their increasing values of elongation at break. The elongation at break increased with increasing TPI contents and then became constant when TPI contents were greater than 10%. Furthermore, the tensile and flexural strength were also enhanced by around 10% by the inclusion of TPI without compromising tensile and flexural modulus. The mechanical properties of semi-IPNs were dictated by several factors including morphology and crosslinking density. At low TPI contents (≤10%), the TPI phases in TPI were discontinuous, and the crosslinking densities remained comparable to pure polycyanurate. The dispersed thermoplastic phases acted as energy absorbers and crack stoppers, leading to moderately enhanced mechanical properties. Semi-IPN−15 and Semi-IPN−20 possessed continuous TPI phases and considerably reduced crosslinking densities, accounting for considerably improved toughness. Due to its lowest crosslinking density, Semi-IPN−20 showed mechanical properties inferior to that of Semi-IPN−15. Generally, Semi-IPN−15 showed the best mechanical properties among all the semi-IPNs because of its optimum crosslinking density and morphology.

#### 3.2.6. Dielectric Properties

**Figure 8:**
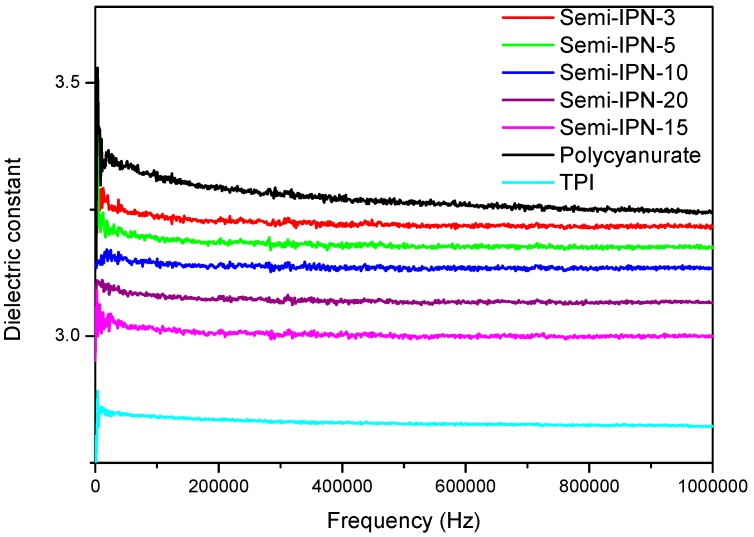
Dielectric constant as a function of frequency for TPI, polycyanurate, and semi-IPNs.

Owing to the presence of low-polar C–F bonds and its rigid but non-coplanar architectures, TPI displayed relatively lower dielectric constant and loss factor in comparison with polycyanurate. Consequently, the dielectric properties of semi-IPNs were improved by the incorporation of TPI, reflected by reduced dielectric constants and loss factors (Table 2 and Figure 8). Moreover, a correlation was observed between the dielectric properties and TPI contents, further confirming the positive role of TPI in the dielectric properties of the resultant polymer networks.

## 4. Conclusions

In summary, five semi-IPNs were prepared via blending BADCy and TPI without the aid of any solvents, followed by trimerization of –OCN functionality. Compared with pure BADCy, TPI/BADCy blends exhibited improved processability, as evidenced by lower viscosity onset temperature and reduced gelation time at 200 °C. SEM results indicated that phase-separated morphologies and continuous TPI phases were formed in semi-IPNs with a high TPI loading (≥15%). The mechanical and dielectric properties of the resultant thermosets were significantly enhanced by the inclusion of TPI without compromising their thermal resistance. Semi-IPNs displayed *T_g_* of 308–312 °C, impact strength of 22–63 KJ·m^−2^, flexural strength of 156–172 MPa, tensile strength of 78–85 MPa, and dielectric constant of 3.00–3.22. These improvements can be explained by the unique characteristics of TPI, which possesses the combined properties of high *T_g_*, excellent dielectric properties, high toughness, and optimum compatibility with BADCy. The blends and semi-IPNs developed in this work could be potentially utilized as high-temperature matrices and adhesives due to their outstanding overall properties and favorable processability. This work came up with new insights into designing high *T_g_* toughening agents and paved ways for further investigation of modified CE resins.

## Figures and Tables

**Table 1:** Cure of BADCy and TPI/BADCy blends.

Formulation	*T_p_* (°C) ^a^	Δ*H*_0_ (J g^−1^) ^b^	Gelation Time (min) ^c^	Viscosity Onset Temperature (°C) ^c^	Isothermal Viscosity (Pa s) ^d^
BADCy	324	709	-	286	0.030
Blend−3	315	648	131	283	0.039
Blend−5	310	600	116	263	0.122
Blend−10	305	565	49	245	1.600
Blend−15	280	557	25	238	3.113
Blend−20	273	469	11	218	6.366

^a^ Peak temperature of differential scanning calorimetry (DSC) curves. ^b^ Heat of cure by DSC. ^c^ Measured by rheometer at 200 °C. ^d^ Measured by rheometer at 100 °C; the viscosity was taken at a frequency of 100 s^−1^.

**Table 2:** Thermal and dielectric properties of polycyanurate, TPI, and semi-IPNs.

Thermosets	*T_g_* (°C) ^a^	*T*_5%_ (°C) ^b^	Dielectric Constant ^c^	Dielectric Loss ^c^
Polycyanurate	310	422	3.25	0.0077
Semi-IPN−3	308	419	3.22	0.0070
Semi-IPN−5	310	421	3.18	0.0060
Semi-IPN−10	312	425	3.13	0.0054
Semi-IPN−15	311	429	3.07	0.0052
Semi-IPN−20	311	428	3.00	0.0050
TPI	360	550	2.85	0.0050

^a^ Measured by DMA with a heating rate of 3 °C min^−1^. ^b^ Measured by thermogravimetric analysis (TGA) in air with a heating rate of 10 °C min^−1^. ^c^ Measured by impedance analyzer; the values were taken at 8 × 10^5^ Hz.

**Table 3:** Mechanical properties of polycyanurate, TPI, and semi-IPNs.

Thermosets	Tensile Strength (MPa)	Tensile Modulus (GPa)	Elongation at Break (%)	Flexural Strength (MPa)	Flexural Modulus (GPa)	Compression Strength (MPa)	Impact Strength (KJ m^−2^)
Polycyanurate	76	3.0	1.8	149	3.2	155	15
Semi-IPN−3	78	3.2	2.2	156	3.3	150	22
Semi-IPN−5	82	3.1	3.1	170	3.1	153	26
Semi-IPN−10	84	3.2	3.1	169	3.2	152	32
Semi-IPN−15	85	3.2	3.1	172	3.2	156	63
Semi-IPN−20	80	2.9	2.9	165	3.0	150	49
TPI	110	2.5	9.3	150	3.0	190	90
